# Theory in qualitative research: a qualitative study of research experts’ views

**DOI:** 10.1186/s12874-025-02752-6

**Published:** 2026-01-08

**Authors:** Oliver Rudolf Herber, Julie Taylor, Caroline Bradbury-Jones

**Affiliations:** 1 School of Nursing Science, Faculty of Health, Witten/Herdecke University, Alfred-Herrhausen-Str. 50, Witten, 58455 Germany; 2 Department of Nursing and Midwifery, School of Health Sciences, College of Medicine and Health, University of Birmingham, Edgbaston, Birmingham, UK

**Keywords:** Application of theory, Interviews, Qualitative, QUANTUM typology, Reporting, Research, Theoretical framework, Theory

## Abstract

**Background:**

There are long-standing debates about the relationship between theory and qualitative research and how the two are connected. While some researchers argue that theory in qualitative research is often superficial and poorly reported, others have highlighted inconsistencies in the application of theory in qualitative research, with it frequently being absent. We had previously developed a typology (known as QUANTUM) in discussing these issues, and wanted to use this as a springboard from which to engage in further debate.

**Methods:**

We engaged with an international panel of 14 qualitative research experts to elicit their views on the place of theory in qualitative research. In a two-stage process, the expert panel provided written comments on the QUANTUM typology and then took part in an interactive, online workshop. Data generated from both stages were analysed thematically. An adapted version of the ‘Five Ways of Knowing’ was used as the theoretical framework for structuring the inductively derived thematic findings in a post-hoc fashion.

**Results:**

The analysis led to the conceptualisation of five themes that were organised according to the components of the ‘Five Ways of Knowing’, namely: (1) Defining Theory; (2) Working with Theory; (3) Positioning Theory; (4) Researchers’ Underlying Epistemological Assumptions; and (5) Problematising Theory.

**Conclusions:**

The themes are discussed against the backdrop of current literature on the topic, capturing the complexity of theory in qualitative research. The input received from the experts facilitated the development of the revised QUANTUM typology, which provides a reflexive aide for the conduct and reporting of theory in qualitative research.

## Background

Several researchers have written about the complex relationship between qualitative research and theory [1–5]. Thorne [6] observed that the place of theory in qualitative research is both heterogeneous and complicated. There are longstanding debates about the relationship and linkage between theory and qualitative research [7]. O’Leary et al. [8] argued that theory in qualitative research is often superficial and poorly reported. In a similar vein, other researchers have reported inconsistencies in the application of theory in qualitative research, with it frequently being absent [9, 10]. Some have argued that the landscape has become fragmented [11].

There are different ways of defining and working with theory and we particularly favour Collins and Stockton’s [12] explanations, whereby a theory is a big idea that organises many other ideas. Unlike a theory of method, which provides guidance regarding the methods, a conceptual framework is a map that illustrates how all the literature cited within a study is interconnected. In a study, a theoretical framework is to use a theory or theories that are explicitly outlined or explained in order to elucidate how new knowledge will be processed [12]. In our own use of theory, we tend to err towards the latter description as being the theoretical lens through which one approaches their qualitative study.

In 2020, we undertook a project to critique and revise an existing typology on the levels of theoretical visibility [9]. In this early version of the typology, we identified five different levels of theoretical visibility, ranging from “seemingly absent” (Level 1), “applied” (Level 2), “partially applied” (Level 3), “retrospectively applied” (Level 4) to “consistently applied” (Level 5). The latter exhibits the strongest link between theory and qualitative research, meaning that the theory serves as the driving force behind the entire study and is applied consistently throughout the entire research process [9]. This research was part of a larger study funded by the Wellcome Trust in the United Kingdom, known as the Qualitative Network for Theory Use and Methodology (QUANTUM). The involvement of 14 international qualitative research experts drawn from health and social science fields was a key element in the project. The aim of this article is to present the experts’ views that were discussed and debated during the workshops regarding the place of theory in qualitative research in general and the typology in particular. This was done with the aim of adapting and revising the existing typology to provide an educational resource (teaching aid) for novice researchers that can raise quality standards for the visibility and description of reporting theory in qualitative research.

## Methods

### Participants

We invited 14 experts in qualitative research (eight from the UK, four from the USA, one from Germany and one from Austria) to participate virtually in this project, all of whom have published extensively (i.e. textbooks, journal articles, etc.) on the subject of qualitative research. Sociology, medical sociology, social policy, nursing, applied psychology, applied health research, physical education and sport, and criminology were among the disciplines represented on the panel. All experts are renowned qualitative methodologists with many years of research experience and specific methodological expertise comprising phenomenology, qualitative content analysis, document and discourse analysis, qualitative meta-synthesis and meta-ethnography. Besides, they take a particular interest in focus groups, sample size estimation in qualitative research, participant observation, interviewing techniques and innovative qualitative research methods. They apply qualitative research to investigate public health issues, use robust qualitative methodologies to engage underserved populations in sensitive health-related research, balance methodological rigour with practical realities, use interpreters and translators in qualitative research, or integrate qualitative studies into large randomised controlled trials. The experts have written numerous articles and textbooks on qualitative research methods and guidelines for reporting qualitative research, or serve on editorial boards of qualitative research journals. The participating experts were identified through our extensive national and international research networks and selected on the basis of their merits in qualitative research.

### Procedure and data collection

In light of the difficulties presented by the COVID-19 pandemic, our original intention to conduct a workshop spanning two days in person had to be reconsidered. Consequently, we resorted to leveraging digital tools such as email and Zoom to facilitate the workshop remotely. This comprised two stages. During the first stage, each member of the expert panel was sent an email containing the original published article on the typology from the 2014 version [9], which contains detailed descriptions of the authors’ conception of the theory. In addition, the email included a link to a recorded slideshow presentation that offered relevant background information on the project, along with a solicitation for written feedback regarding the topics outlined in Table 1. In April 2020, all members of the panel responded to our call and returned written feedback via email. Stage two took place in May 2020, where we conducted two interactive workshop sessions via Zoom, each lasting for about 90 min. Approximately half of the expert panel participated in each session. We divided them in this manner to enhance interaction within a smaller group, thereby providing more opportunities for contributions. The purpose of the sessions was to foster additional dialogue regarding potential modifications to the existing typology, establish a platform for experts to convene and exchange ideas, and engage in thoughtful deliberation on the issues that were brought up. Participants gave verbal consent to use Zoom’s recording facility to capture the online workshops verbatim.

**Table 1:** Request for written feedback from qualitative research experts on the points of consideration

The purpose of the QUANTUM Typology is to provide an educational resource/teaching aid through which the relationship between theory and qualitative research can be understood. The following questions were asked of the participating experts:	
• To what extent does the Typology achieve this purpose? Please give reasons. • In the article the five levels of visibility indicate the levels at which theories are made explicit within a sample of academic publications. What are your thoughts regarding the five levels? • Can you think of other levels of visibility? • How might the five levels be improved? • What resources/tools, if any, do you currently use to review the use of theory when peer reviewing articles or supporting students and researchers in their report writing? • How, for example, do you gauge the clarity with which theory is articulated and reported? • How might the Typology be used by researchers in the writing process? For example, are the five levels transferable from judgements about publications to the work of student researchers? • How might the Typology be used by external experts involved in quality assurance at specific stages of the research process? • Could the typology be added as a separate criterion to existing reporting guidelines for qualitative research? • What are the limitations and shortfalls of the Typology? What we need from you now: • Please share your feedback/ideas regarding the Typology that respond to the points that we have posed. • We really welcome comments on anything that has not been covered in our questions.	

During these two phases, we gathered a substantial amount of invaluable data that went well beyond the information that we required to revise the typology. It is this rich data that we report on in this article. Feedback on the typology from the first stage was sent by email, consisting of 23 pages (A4 size) of comments, annotated articles and suggestions from several experts. In the second stage, we have produced a 180-minute Zoom recording of the online workshops including the accompanying text format of the Zoom ‘chat’ boxes. Unexpectedly, the compulsory utilisation of digital technologies due to COVID-19 has produced a significantly larger amount of data than we had initially expected.

### Ethical considerations

To allow us to do it justice and turn ‘information’ into ‘data’, we applied for retrospective ethical approval for the study, which was granted by the Science, Technology, Engineering and Mathematics Ethical Review Committee of the University of Birmingham (ethical approval number: ERN_20–0789). Written informed consent to make use of all provided information was obtained retrospectively via email by CBJ from all research experts. Participants were assured confidentiality of all provided information. The research was conducted according to the World Medical Association Declaration of Helsinki [13].

### Data analysis

An inductive approach was utilised to thematically analyse the data generated during the two stages [14, 15]. The analysis was led by CBJ, and subsequently, the identified themes were deliberated upon and reached a consensus with the rest of the team members. This cross-verification corresponds to the concept of intercoder agreement as described by Campbell et al. [16]. In this process, two or more coders resolve any differences in coding by engaging in discussions. We regarded it as a crucial measure to strengthen the rigour of the analysis. Every expert was allocated a unique code to safeguard their confidentiality. The majority of quotations displayed in the findings section are derived from the email feedback, bolstered by data obtained from the online workshops.

### Theoretical framework

As already mentioned, we had not begun the project with the intention of generating qualitative data. Instead, it came about as an unexpected by-product of the considerable feedback we had received from the expert researchers who engaged with the project. Our thematic findings as presented in the following section were derived inductively. It was not until the thematic patterning, at an advanced stage of the process, that a theoretical lens through which to capture the findings began to infiltrate the consciousness of the co-author CBJ undertaking the analysis. According to the existing typology on the levels of theoretical visibility [9], this approach could be classified as “retrospectively applied” (Level 4), where theory is considered at a later stage of the study as a means of making sense of the research findings.

Carper’s [17] seminal work on fundamental patterns of knowing in nursing proposed four ways through which nurses learn: empirical, aesthetic, personal and ethical. It has been expanded upon over the years into full works, notably the comprehensive textbook of Chinn et al. [18]. An important component of the expansion has been the integration of a fifth pattern: The Emancipatory Way of Knowing [19, 20]. We therefore used Carper’s four fundamental ways of knowing in nursing and Chinn et al.’s fifth, emancipatory, as our theoretical framework for structuring our inductively derived thematic findings in a post-hoc fashion. The left-hand column shows the original understanding of the ‘Five Ways of Knowing’ and these are contrasted with how we applied the descriptors to the current study in the right-hand column (Table 2).

**Table 2:** The five ways of knowing related to theory and qualitative research

Way of Knowing	Original meaning	Application to current study
Empirical	The science of nursing which is factual, descriptive and informed by research and firm evidence.	How theory is understood in scientific terms. => Defining Theory
Aesthetic	The art of nursing that involves an intuitive sense of patients’ needs. It is a tacit knowledge that takes account of nuanced information.	Ways in which theory can be used creatively. => Working with Theory
Ethical	Ethical knowing in nursing is based on the morals associated with choices. It is informed by judgements about what is right and wrong.	Moral judgements about how best to use (or not use) theory. => Positioning Theory
Personal	The personal way of knowing encompasses experiential learning. It draws upon the knowledge we have of ourselves and how these shape our interactions and behaviours.	An individual researcher’s lens and the influence this has on the way theory is used. => Researchers’ Underlying Epistemological Assumptions
Emancipatory	Emancipatory knowing advocates for social justice and human rights on behalf of others. It challenges inequalities and disparities.	The need to challenge theoretical hegemony and provide space for marginalised voices. => Problematising Theory

## Results

The analysis led to our conceptualisation of five themes that are organised according to the components of the ‘Five Ways of Knowing’, namely: (1) Defining Theory; (2) Working with Theory; (3) Positioning Theory; (4) ‘Researchers’ Underlying Epistemological Assumptions and (5) Problematising Theory.

### Theme 1: defining theory

Defining Theory deals with the question of how theory is understood by the experts in their scientific field. The valuable contributions of the experts have undoubtedly enriched our understanding, distinguishing between different theories and providing insightful definitions of theory in the context of their own qualitative research:

*“A useful way of looking at it is the distinction between grand theories (overarching conceptual theories*,* unified theories about the social world)*,* mid-range theories (a patterned set of hypotheses or assumptions about how things in a focused area are connected)*,* and programme theories (small theory about how a policy or programme is intended to work). It also*,* I think*,* assumes that theory is a lens that is brought to bear on the study*,* rather than the subject of the study itself.”(Expert 4)*.

The following quote provides a further distinction between the theories that underpin the methodology and the substantive theories. It notes that students, particularly those new to research, may find it difficult to critically evaluate substantive theories and may be inclined to accept them unquestioningly:

*“Theories underpinning methodology and substantive theories underpinning their subject area and how to critique the latter systematically through their work. This is something that I find students can struggle with. Students who are new to research can simply accept substantive theory without question.”(Expert 6)*.

Another expert suggested four theoretical elements that are the hallmarks of ‘a good qualitative study’:

*“We discriminate four areas of theory behind a research project: A: Object theory: State of the art in respect to object and research question(s); B: Discipline theory: General approach to the discipline in respect to all objects (e.g. psychoanalytic*,* behaviouristic*,* cognitive); C: Methodological Theory: Theoretical arguments for the choice and construction of research methods used in the study; D: Theory of Science position (constructivist*,* postpositivist*,* pragmatic*,* critical). A good qualitative study should explicate the theoretical basis in all four areas.”(Expert 14)*.

### Theme 2: working with theory

Working with Theory looks at the different ways in which theory can be used in a creative way. A number of experts talked about the implicit or explicit use of theory and the visibility of theory within a research report, as illustrated by expert 4:

*“I strongly endorse the line taken that all research involves theory and that a key issue is how far this is explicit and visible. My gauge of the clarity with which theory is articulated would be pretty intuitive – whether what’s been said has surface validity*,* i.e. I can understand it*,* it seems logical*,* I can see how it’s enhanced the study*,* it’s informed all stages (research questions / understanding of them*,* sampling*,* issues addressed*,* analysis*,* reporting – and it’s informed not just what is observed*,* but informed how we’ve looked at the relationships between things: the causes*,* mechanisms*,* moderators*,* mediators etc.).”(Expert 4)*.

The potential for multiple theories to be drawn upon in one study was also discussed as a way of working with theory in qualitative research:

*“There might be numerous types of theories involved in a single paper*,* where it would be appropriate to only briefly touch on part of a theory.”(Expert 10)*.

Another researcher takes a similar view, with reference to the multiple theories that are at play in her research on social interventions and their visibility:

*“I think often multiple theories are at play*,* and it’s important to make them all visible. Most of my research is applied to the study of social interventions and much of it is commissioned*,* so for me there would be multiple theories at play around: the theory/ies underpinning the intervention; those of the organisation delivering it [very implicit and hard to surface]; those of the research funder [ditto]; those I [on a good day! ] explicitly bring to the study from the start; any further theory/ies I might bring in specifically to make sense of the findings.”(Expert 4)*.

### Theme 3: positioning theory

Positioning Theory refers to the moral judgements about how best to use (or not use) theory. Many research experts talked about the iterative nature of theory in qualitative research as illustrated by experts 8 and 3:

*“The relationship between theory and research*,* can be iterative and two-way*,* and involves a process of understanding and application.”(Expert 8)*.

Several experts emphasised that qualitative research is an iterative process with theories entering and exiting at different stages, acknowledging the challenge of capturing the dynamic evolution of theories:

*“Much of qualitative research is iterative*,* so theory can come in at various stages in the research*,* or theory may enter and exit*,* or theory might start quite broad (as a general orientation) and become more specific. ‘Theory life’ or ‘story of the theory’ is difficult to convey in a research paper.”(Expert 3)*.

The experts had a great deal to report on where theory comes into play in qualitative research. This was articulated around three domains: beginning, evolving and infusing. Many of the experts talked about the importance of beginning with a theory and as articulated by expert 1, using it as a ‘sensitising’ framework:

*“Maybe think about the influence of theory on the research act. As ‘sensitising’ or creating the framework for the performance of the research.”(Expert 1)*.

One expert questioned whether a qualitative study always has to start with a pre-defined theory, particularly in the field of health professions education, where there is an expectation that high-quality studies present a conceptual framework:

*“Does a ‘good’ qualitative study always need to have an identified theory upfront guiding the study? In my field (health professions education) there are strong messages that high quality studies present a conceptual framework upfront.”(Expert 3)*.

The experts emphasised the importance of integrating theory into the design stage of research studies to ensure that theoretical frameworks drive and inform the work. Expert 9 points out that asking for details of theory use in publications could encourage researchers to consider theory during the design process:

*“Theory should be used at the stage of designing a study. If we want theory to drive and inform our thinking and our work*,* that is where it needs to be*,* although I realise if we start to ask for details of theory use in publications*,* this might then encourage researchers to consider theory when designing their research.”(Expert 9)*.

A prominent point made by the experts was in relation to the frequency of theory coming to light *during* a study, as opposed to the beginning, with a legitimacy in such evolving. This was particularly the case with some methodologies, for example phenomenology and grounded theory:

*“I have seen many people not know what theory to use until they get their data analysed and then one becomes apparent. Grounded theory and phenomenology are supposed to be atheoretical. Of course*,* I will not go back into the history and development of these two methodologies*,* but there have been long-standing traditions supporting this. I do very much appreciate someone finally articulating this*,* after 30 + years of conducting and guiding qualitative research.”(Expert 7)*.

The following quote highlights the dynamic nature of research, suggesting that unexpected themes and findings may evolve during analysis or reporting, leading to the consideration of theories not initially contemplated:

*“I think a study can result in themes and findings not initially considered*,* which then results in theories not previously considered appearing relevant at the analysis or reporting stage. For example*,* I remember a colleague doing a piece of work in child’s homes. She then realised there were different types of power*,* e.g. knowledge*,* finances*,* and then drew on Bourdieu’s concept of cultural capital to interpret her findings.”(Expert 8)*.

Within the experts’ quotes, we perceived the notion of ‘infused with theory’. In other words, the expectation that theory might be seen permeating through the work, almost in a capillary fashion:

*“If an author says they are using a theory in their research*,* I expect it to show up in multiple places. I want to see how the theory influenced their thinking at each stage of the research process. This is clarity for me. I am also fine with authors who find a theory post-hoc.”(Expert 7)*.

Some experts stressed the importance of a clear and consistent articulation of theory in different parts of a research paper, with particular attention to the alignment between the introduction, methods, results and discussion of the study:

*“I would expect to see more clarity with which theory had been articulated and reported. I would look for it in the introduction*,* methods*,* results and discussion*,* and check whether there was alignment across these sections in terms of how it was being applied*,* e.g. if the introduction talked about using the Health Behaviour Model to understand behaviours*,* I would then check to see if perceived susceptibility*,* severity etc. were explored during the interviews*,* focused on in the analysis*,* reported in the results and considered in the discussion.”(Expert 8)*.

### Theme 4: researchers’ underlying epistemological assumptions

Researchers’ Underlying Epistemological Assumptions refers to the individual lens of the researcher and the influence this has on the way theory is used. Given that the reflexive use of self and co-production is at the heart of qualitative work, it is interesting to note that only one expert referred to this important component, as the following quote demonstrates:

*“I also wonder how philosophical orientations fit in. For example*,* in my field (health professions education) there has been much attention recently to the importance of scholars stating their epistemological and ontological orientation (e.g. constructivist*,* port-positivist*,* etc.).”(Expert 3)*.

### Theme 5: problematising theory

Problematising Theory addresses the need to challenge theoretical hegemony and make space for marginalised voices. As exemplified in the opening quote for this section, we were not surprised to see a significant degree of problematisation among the experts, who put forward multiple, critically reflective viewpoints about theory in qualitative research:

*“If theory becomes a replacement for robust research rather than an aid (paraphrasing Silverman*,* 2007)*,* then it is problematic.”(Expert 1)*.

A fundamental issue is choosing the ‘right’ theory. As illustrated by expert 4, the subsequent quote prompts critical reflection on the appropriateness of the chosen theory by questioning its effectiveness, potential limitations, impact on interpretation, and considering alternative theories:

*“Was it the right theory to have used? What did it illuminate? What might it have obstructed? How might it have led the interpretation in a particular direction? What other theories might have been used?”(Expert 4)*.

The ‘right’ theory then needs to be well understood and utilised appropriately:

*“It’s all well and good to clearly articulate that you’ve used theory*,* but if the use of theory is poor or methodologically inappropriate then actually is it any better than those studies who use it partially? To me*,* the key is about the authors very clearly articulating how they have used (or not) theory in their research and that this is explicitly contextualised.”(Expert 9)*.

Some experts emphasised that the effective use of theory in research goes beyond mere application and requires thoughtful modification or refinement through empirical investigation, challenging the notion of treating theories as inflexible principles and highlighting that many theories lack empirical testing or support:

*“You could have used theory and done a rubbish job of it – picked a poor theory*,* or operationalised it thoroughly but poorly. The hallmark of good research*,* and good use of theory in it*,* is whether the theory has been usefully modified (or confirmed*,* or disproven) or just better understood as a result of the research. We shouldn’t be treating theory as tablets of stone. Lots of what we regard as theory is not empirically tested*,* let alone empirically supported.”(Expert 4)*.

A prominent narrative among the experts was the notion of ‘constraining theory’. Within this, experts critically engaged with the limitations and shortfalls of having theory ‘up front’:

*“I think this [having a theoretical framework upfront] causes some problems because then authors retrospectively apply a theory or framework that really was not used to guide the study*,* so the alignment is unclear and the theory feels forced (not to mention the authors are misrepresenting how the theory was actually used).”(Expert 3)*.

A few experts questioned whether the theoretical framework used to structure the research may impose limitations on the interpretation of the study’s findings:

*“Does the theory of framing the research actually limit interpretation?”(Expert 1)*.

Furthermore, several experts highlighted a desire for greater acknowledgment of the limitations associated with a theory-driven approach to qualitative research, pointing out the potential drawbacks of solely deductive methods that may overlook data not aligned with the initial theoretical assumptions:

*“I’d have liked to see more acknowledgements of the ‘limitations’ of a theory driven approach to qualitative research; a purely deductive approach can be problematic as it ignores data that fall outside of the initial theoretical premises.”(Expert 6)*.

In a similar vein, experts critically noted that while a theory may shape research questions and analysis, relying solely on the theory’s framework could be both a strength and a limitation:

*“With a discipline-based theory*,* the framing of the research questions and maybe even the structure of the analysis*,* might be driven by the theory*,* but that might actually be a problem as well as a strength*,* as the emerging findings might not be entirely contained in the theory.”(Expert 2)*.

Finally, although raised by one expert only (at least explicitly), we are keen to capture the problem of what we term ‘theoretical hegemony’. Within this, expert 11 called for a critical evaluation of the privileging of certain forms of knowledge and the marginalisation and silencing of others:

*“An ongoing conversation in my home field of […] is examining the “whiteness” of certain strains of literature and concepts. Theory is certainly something that falls into this discussion as scholars often rely on White and Western ideas of what equals theory. As such*,* I think it’s important to consider non-white*,* non-western*,* or indigenous ideas when defining theory.”(Expert 11)*.

We have chosen to end with this quote, not because we think it is less important than any of the others, but because of its relevance to qualitative research. If we allow theoretical hegemony to go unchallenged, then the way we define, work with and position theory becomes irrelevant.

## Discussion

The following discussion addresses some aspects of the themes presented in the findings section according to the modified descriptors of the ‘Five Ways of Knowing’. The discussion commences with the theme of ‘Defining Theory’, followed by ‘Working with Theory’, ‘Positioning Theory’, and ‘Researchers’ Underlying Epistemological Assumptions’. This latter theme, to our surprise, received hardly any feedback from our experts. The critical issues of reflexivity, power and privilege, as highlighted by global experts from (non-) Western countries, were overlooked by our experts in their analysis of the current global body of qualitative theoretical thinking. In light of the absence of these perspectives, we feel compelled to offer some insights into the debate surrounding theoretical hegemony and the problematic nature of Western humanism as a universalist conception. The discussion concludes with a focus on the theme of ‘Problematising Theory’.

### Defining theory

While there are a plethora of definitions as regards meaning of theory, one of our experts mentioned that theories can have different levels of abstraction. A distinction can be made between the theories in terms of their explanatory power and level of abstraction [21]. Grand theories – also referred to as broad-range theories – are highly abstract, tend to be concerned with broad natural and social patterns like human needs (e.g. Orem’s Self-Care Deficit Theory of Nursing; [22]) and because of their scope and level of abstraction, they do not lend themselves readily to testing. Unlike grand theories, middle-range theories have a limited scope and less abstraction and are focused on a particular part of a discipline’s concerns (e.g. Leininger’s Theory of Culture Care Diversity and Universality; [23]). The narrower scope and specificity of a middle-range theory make it easier to use and test in research. Microtheories or situation-specific theories are described as practice theories that give a description, explanation, and understanding of individual-level phenomena (e.g. Herber’s Theory of Barriers and Facilitators for Self-Care in Patients with Heart Failure; [24]). In order to choose the most appropriate theory for their research, researchers have to do a lot of reading and even so, as our expert group showed, may still disagree about what defines ‘theory’.

### Working with theory

Several experts endorsed the line taken that all research involves theory and that a key issue is how explicit the use and visibility of theory within a research report ought to be. Researchers should neither be atheoretical nor start their research with a single theoretical framework [18]. The possibility of using multiple theories in a single study, as mentioned by some of our experts, is consistent with the disciplinary epistemological orientation of (for example) nursing researchers, where subjective knowledge is valued and the potential for multiple realities exists [25]. Therefore, adherence to a particular theory or theoretical framework in a practical and applied discipline such as nursing would be incongruent with the way nurses understand the world [6]. Nursing phenomena related to complex patient care cannot be meaningfully studied by limiting nurses’ decision making to a single theoretical perspective. Instead, they would draw on previous practice experience, various sources of evidence and multiple theoretical perspectives to assess and interpret a situation [25]. Thorne [6] highlights that during the iterative process of data collection, analysis and interpretation applied practice researchers are encouraged to explore a number of potentially relevant theoretical ideas. If researchers remain open to a wide variety of theoretical positions, they will not only avoid privileging certain theoretical positions over others, but also prevent missing out on the critical value of contextual applied understandings when analysing emerging data [26].

### Positioning theory

There was some discussion among our experts about the iterative nature of theory in qualitative research and how theory can enter or exit at different stages of the research process. We believe that all knowledge is theory laden, and therefore the use of theory should be explicitly described in qualitative research [12]. However, Nguyen et al. [27] believe that applying a theory throughout the research process by following a rigid typology is fruitless. This is in line with Shelton et al. [28], who advise researchers to adopt a critical, yet flexible and creative way of applying theory in qualitative research.

In addition, a few experts questioned whether a qualitative study always needs to have an identified theory at the outset, noting that in some professions there is a strong message that high quality studies present a conceptual framework upfront. According to Thorne [6], there is a widespread belief in nursing that a study must be conceptually grounded in a theoretical framework from the outset, unless methodological positioning explicitly requires a researcher to enter a study with a ‘blank slate’ (*tabula rasa*). Over time, this belief has evolved into a commonly accepted quality indicator and reporting requirement [29], reinforced by quality appraisal tools and journal guidelines that seem to associate the use of theory or theoretical framework with a higher level of coherence. While this belief may still hold true for conventional social science methodological traditions such as grounded theory, ethnography or phenomenology where the focus is largely on developing and refining theory, it must be critically challenged when it comes to applied (‘generic’) qualitative research methodologies where the primary purpose is to generate insights and knowledge that can be applied to nursing practice [25]. According to Chiu et al. [25], research calls for a different relationship with theory by making explicit justifications for why research should neither be atheoretical nor conducted using a single theoretical framework at the outset. Instead, researchers are encouraged to explore a range of potentially relevant theories or theoretical frameworks to draw on iteratively during data collection, analysis and interpretation [6].

During the interactive workshop session, some experts mentioned the long-standing debate about the atheoretical nature of grounded theory and phenomenology and the desire, finally, to articulate this. We are hereby making an attempt to do so. Over the years, philosophical debates have contributed to the emergence of intriguing variations in the use of theory in grounded theory and phenomenology. For example, while the original (Glaserian) approach to grounded theory [30, 31], influenced by positivism, required researchers to enter the study without the use of theory, Charmaz’s [32] approach, grounded in social constructivism, suggests an implicit use of theory. Similarly, a range of variations can be illustrated for phenomenology. Transcendental (or Husserlian) phenomenology, based on the idea that reality is internal to the knower [33], requires researchers to ‘bracket’ their preconceptions in order to discover it. As a result, explicit or implicit use of theory is incompatible with this perspective. On the other hand, hermeneutic (or Heideggerian) phenomenology, rooted in the assumption that lived experience is not separate from the world, allows researchers to incorporate theory in order to inform design decisions [34].

### Researchers’ underlying epistemological assumptions

It is somewhat surprising that the issue of the researcher’s lens and its influence on the way theory is used was not really considered by our experts. Global experts from (non-)Western backgrounds are increasingly, and rightly, arguing that a lack of critical engagement with key issues such as reflexivity, power and privilege has resulted in a lack of reflection on the current state of qualitative theoretical thinking. For example, a growing body of critical literature has emerged that raises concerns about the concept of Western humanism as a universalist concept. The core assumptions of Western humanism place white, heterosexual, and able-bodied males at the pinnacle of a hierarchical structure, thereby obscuring the experiences of marginalised women and people of colour [35].

### Problematising theory

Several experts raised a fundamental issue concerning the use of theory in qualitative research, namely the rationale for and selection of the ‘right’ theory or theories. Considering that epistemological and ontological dispositions influence how a researcher sees the world and produces knowledge, selecting a qualitative research theory is strongly influenced by these dispositions. Glesne [36] emphasises that epistemology influences the selection of theory and the degree to which the two are working together in the analytic approach. In summary, it can be stated that epistemology influences theory selection, which makes the two mutually interdependent [12].

Another vital issue raised by one expert relates to what we term theoretical hegemony, i.e. the privileging of certain forms of knowledge to the marginalisation and disempowerment of other. Indigenous communities are increasingly calling for research to be decolonised, i.e. to question the underlying assumptions that inform research and to challenge Western methods as the only ‘true’ science by placing Indigenous voices and epistemologies at the centre of the research process [37, 38]. This does not mean that all Western methods and theories should be rejected at the outset, as long as they can be adapted to be appropriate and beneficial to the local community. However, if we continue to use monocultural Western research paradigms, mainstream voices will be prioritised and Indigenous voices will be excluded from academic discourse, further marginalising communities [39]. Therefore, in terms of implications for research practice, we urge qualitative researchers to contemplate the consequences of perpetuating Western and Global North ways of knowing as superior, as this can result in the marginalisation and suppression of Global South and Indigenous knowledge systems.

### Strengths and limitations

In terms of strengths, the themes identified during the analysis were discussed with the rest of the team and consensus was reached. This process of cross-checking is consistent with the concept of intercoder agreement [16], where multiple coders resolve discrepancies in coding through discussion. We considered this as an important measure to strengthen the accuracy and reliability of the analysis. Another strength was the involvement of national and international qualitative research experts from multiple disciplines, who provided valuable insights into the use of theory from different perspectives. In terms of limitations, we have essentially been working with secondary data because we utilised the abundance of rich information provided by the panel that was not generated with our purpose in mind. To that end, the data may not be as complete as they may have been if we had set out to conduct the study with primary questions to be answered. However, we consider that the overall analysis has potential to be of use to qualitative researchers (particularly novice and early career researchers) who are provided with a new way of understanding and framing the inherent complexities within qualitative research. Moreover, the differences in viewpoints between our experts could have been grounded in their differing views regarding definitions of theory, but we did not manage to scratch below the surface in exploring this. A further limitation is the exclusive involvement of experts from Westernised countries in our panel, which has resulted in a lack of critical reflection in relation to Theme 4 ‘Researchers’ Underlying Epistemological Assumptions’.

## Conclusions

The purpose of this article is to present the experts’ views on the place of theory in qualitative research. We have proposed five themes to consider when capturing the complexity of theory in qualitative research. The intention was to update and modify the current typology in order to create a teaching tool for novice researchers that aims to raise quality standards in terms of presenting and describing theory in qualitative research. The input received from the experts facilitated the development of a new, revised typology – known as the QUANTUM typology, which has been described in detail elsewhere [40].

## Data Availability

The datasets generated during the current study are not publicly available due to securing the individual privacy and confidentiality of the participants. Data are available from the corresponding author on reasonable request.

## Abbreviations

QR-LAW: qualitative research level of alignment wheel ™
QUANTUM: Qualitative network for theory use and methodology
